# Work ability and psychological distress in a working population: results from the Stockholm Public Health Cohort

**DOI:** 10.1177/14034948211033692

**Published:** 2021-08-21

**Authors:** Clara Onell, Lena W. Holm, Tony Bohman, Cecilia Magnusson, Mats Lekander, Eva Skillgate

**Affiliations:** 1 Musculoskeletal & Sports Injury Epidemiology Center, Department of Health Promotion Sciences, Sophiahemmet University, Sweden; 2 Unit of Intervention and Implementation Research for Worker Health, Institute of Environmental Medicine, Karolinska Institutet, Sweden; 3 School of Health and Welfare, Dalarna University, Sweden; 4 Department of Global Public Health, Karolinska Institutet, Sweden; 5 Department of Clinical Neuroscience, Karolinska Institutet, Sweden; 6 Department of Psychology, Stress Research Institute, Stockholm University, Sweden

**Keywords:** Epidemiology, occupational health, work ability, psychological distress

## Abstract

**Aims:**

Psychological distress is a global public health concern with individual and societal implications causing work-related disability and loss of productivity. It is less known how much work ability contributes to the development of psychological distress. This study aimed to assess the association between self-perceived physical and mental work ability in relation to job demands, and the incidence of psychological distress in a Swedish working population.

**Methods:**

Data were obtained from three subsamples of the Stockholm Public Health Cohort with baseline in 2010 and follow-up in 2014, based on a working population in Stockholm County aged 18–60 years, with no or mild psychological distress at baseline (*n*=29,882). Self-perceived physical and mental work ability in relation to job demands were assessed at baseline with a subscale from the Work Ability Index. Study participants scoring 4 or more on the General Health Questionnaire 12 at follow-up were classified as having developed psychological distress during the study period. Poisson log linear regression was used to calculate crude and adjusted rate ratios with 95% confidence intervals.

**Results:**

At follow-up, 2543 participants (12%) had developed psychological distress. Reporting poor physical and/or poor mental work ability in relation to job demands at baseline was associated with an almost doubled rate ratio of psychological distress at follow-up, compared to reporting good work ability (rate ratio 1.8; 95% confidence interval 1.6–2.0).

**Conclusions:**

**Poor work ability is associated with a higher incidence of future psychological distress compared to good work ability.**

## Background

Mental health problems are leading causes of years lived with disability in high and middle-income countries. Depression and anxiety are most common within this wide scope of diagnoses, with an estimated total global prevalence of 7%, according to the Global Burden of Disease Study [1]. In Sweden, mental health problems are the primary reason for long-term sick leave, which, apart from personal suffering, is associated with a great societal burden and loss of productivity [2]. Psychological distress (PD) is characterised by a continuum of depressive and anxiety symptoms, which may develop into mental illness with greater severity if unrecognised. Notably, a systematic review of prospective studies and a longitudinal study report that subthreshold depression is associated with future major depressive disorder as well as other psychiatric conditions [3, 4].

Working conditions promoting physical and mental health are of importance for a sustainable and long-lasting working career, including prioritising primary prevention of occupational health hazards such as PD. Previous studies have examined the role of psychosocial factors in work environments for future mental health. In a meta-review by Harvey et al. (2017), low job control, high job demands, low relational justice, high effort–reward imbalance, role stress, bullying, low procedural justice and low social support were identified as risk factors for depression and anxiety symptomatology [5]. Similar findings were presented in a systematic review by Theorell et al. (2015) presenting low decision latitude, job strain and bullying as risk factors for depressive symptoms [6]. Furthermore, Madsen et al. (2017) concluded in a systematic review that job strain was associated with not only depressive symptoms but also an increased risk of clinically diagnosed depression [7]. Evidently, work-related factors are important determinants for mental health.

Work ability is defined as having occupational competence and health to manage reasonable tasks in an acceptable working environment [8]. Assessing work ability in occupational settings is valuable to recognise ill health and prevent workers from discontinuing due to work-related disability [9]. Although measures of work ability ideally include an assessment of both objective and subjective estimation of workers’ own resources in relation to job demands [10], self-reporting instruments such as the Work Ability Index (WAI) are measures with high validity often used in occupational research. The WAI consists of seven subscales measuring current work ability compared to the lifetime best, work ability in relation to job demands, current diagnosed diseases, work impairment due to disease, sick leave during the past year, own prognosis of work ability and mental resources [11]. The WAI is widely used in occupational health research with results that have been proved to be a good predictor of frequent sickness absence [12] as well as future work-related disability and sick leave [13–15]. Moreover, Ebener and Hasselhorn (2019) concluded that measuring work ability with only the subscale about work ability in relation to job demands of the WAI is feasible in occupational health research [16].

In a systematic review by van den Berg et al. (2009), high physical workload, poor physical work environment, high mental work demands, lack of autonomy, obesity, older age, lack of leisure-time vigorous physical activity and poor musculoskeletal capacity were associated with poor work ability [17]. Leijon et al. (2017) found that PD was associated with decreased work ability in a longitudinal study of Swedish workers [18]. To date, little is known about whether there is also a reversed association; that is, if poor work ability is of importance for the incidence of PD as other psychosocial work-related factors have shown relevance for mental health problems [5–7].

## Aims

The aim of this study was to assess the role of self-perceived physical and mental work ability in relation to job demands for the incidence of PD in a Swedish working population.

## Methods

### Design and study sample

This study is based on data obtained from the Stockholm Public Health Cohort (SPHC), a prospective study set within the framework of Stockholm County Council’s public health surveys aiming to assess health, lifestyle and socioeconomic factors. Further description and details of the SPHC is found in the cohort profile article [19]. A cohort was formed based on three subsamples of the SPHC; one recruited in 2002 with follow-up in 2006, 2010 and 2014, a second formed in 2006 with follow-up in 2010 and 2014 and a third formed in 2010 with follow-up in 2014. In this study, data obtained from the three subsamples of the SPHC surveyed in 2010 and 2014 were merged so that all study participants had the same baseline (2010) and the same follow-up (2014).

Men and women between 18 and 60 years of age who participated in any of the three subsamples of the SPHC were included in the study if they reported no or mild PD at baseline; that is, scoring 2 or less on the General Health Questionnaire 12 (GHQ-12, described below) [20]. Another criterion for inclusion was responding to any of the two items on the subscale about physical and mental work ability in relation to job demands from the WAI (described below); that is, indicating that the participants had an active working life. Those with sickness absence for more than 90 days during the past 12 months were excluded from participation.

### Exposure

At baseline in 2010, physical and mental work ability was measured with the subscale about work ability in relation to job demands included in the WAI. Participants were asked to answer the questions: (a) How do you rate your current work ability with respect to the physical demands of your work? and (b) How do you rate your current work ability with respect to the mental or psychological demands of your work? Physical and mental work ability was merged in which ‘poor work ability’ was defined as answering ‘moderate’, ‘rather poor’ or ‘poor’ in one or both of the items, and ‘good work ability’ was defined as answering ‘rather good’ or ‘very good’ on both items. Previous studies show that the psychometric properties of this subscale from the WAI are considered stable, predictive and internally coherent on group level [21, 22].

### Outcome

The incidence of PD at follow-up in 2014 was assessed with the GHQ-12. Participants were asked to respond to six positive and six negative statements in relation to their psychological health during the past weeks. The positive statements (i.e. ability to concentrate, playing a useful part, capability of making decisions, ability to enjoy day-to-day activities, ability to face problems and feeling reasonably happy) had multiple answer alternatives ranging from ‘more/better than usual’ to ‘much worse/less than usual’. For the negative statements (i.e. loss of sleep over worrying, feeling constantly under strain, incapability to overcome difficulties, feeling unhappy and depressed, losing confidence and thinking of self as worthless), answer alternatives ranged from ‘not at all’ to ‘much more than usual’. A score of 4 or more on the GHQ-12 was defined as having PD at follow-up, in which the chosen cut-off corresponding to moderate and severe PD was based on a previous validation study and is the recommended standard scoring method for PD assessed with this instrument [23]. Several studies have concluded that the GHQ-12 is a valid and reliable instrument in general population samples [20, 23–25].

### Confounding

Potential confounders were considered in accordance with previous research on risk factors for mild mental illness, clinical considerations and availability, after careful discussion about whether they instead could be intermediators or colliders. Potential confounders are summarised in Table I.

**Table I.** Potential confounders.

Variables	Categorisation^a^
Age	Continuous, categorised in 5-year intervals
Sex	Man, woman
Socioeconomic status	Unskilled/semiskilled workers, skilled workers, assistant non-manual workers, intermediate non-manual workers, employed/self-employed professionals, self-employed other than professionals
Body mass index	Continuous, categorised <18.5, 18.5–24.9, 25–29.9, ⩾30 kg/m^2^
Daily smoking	Yes, no
Leisure-time physical activity past 12 months	Walking/cycling/other activities <20 min/day and other activities <1 hour/week, walking/cycling/other activities >20 min/day and other activities >1 hour/week
Household	Living alone, living with children, living with adult(s) with/without children
Neck and/or back pain past 6 months^b^	Yes, no

a Assessed from the Stockholm Public Health Cohort baseline questionnaires. Socioeconomic status retrieved from the National Swedish Registers (combination of occupation and education).

b Neck and/or back pain classified as pain in any area regardless of frequency and severity.

### Statistical analysis

Generalised linear models with Poisson log linear regression were used to estimate the associations between work ability and PD. Results are presented as rate ratios (RRs) with 95% confidence intervals (CIs). Factors considered as confounders in the association between the exposure work ability and the outcome PD were age, sleep disturbance and neck/back pain in the past 6 months, which were added to the final model. Also, a variable including the origin of the three subsamples (i.e. years 2002, 2006 or 2010) were added to the model because the baseline in this study (2010) was not the baseline for two of the subsamples. To assess potential selection bias, an attrition analysis was conducted by comparing the prevalence of poor work ability among those lost to follow-up and those with missing data on any of the outcome variables, with the prevalence of this exposure among those successfully followed. Statistical analyses were performed using IBM SPSS Statistics version 25.

### Ethical considerations

Participants gave their written informed consent prior to data collection. The study was approved by the ethical review board of Stockholm (Dnr 2010/1185-31/1 and Dnr 2016/987-32).

## Results

**Figure 1.**
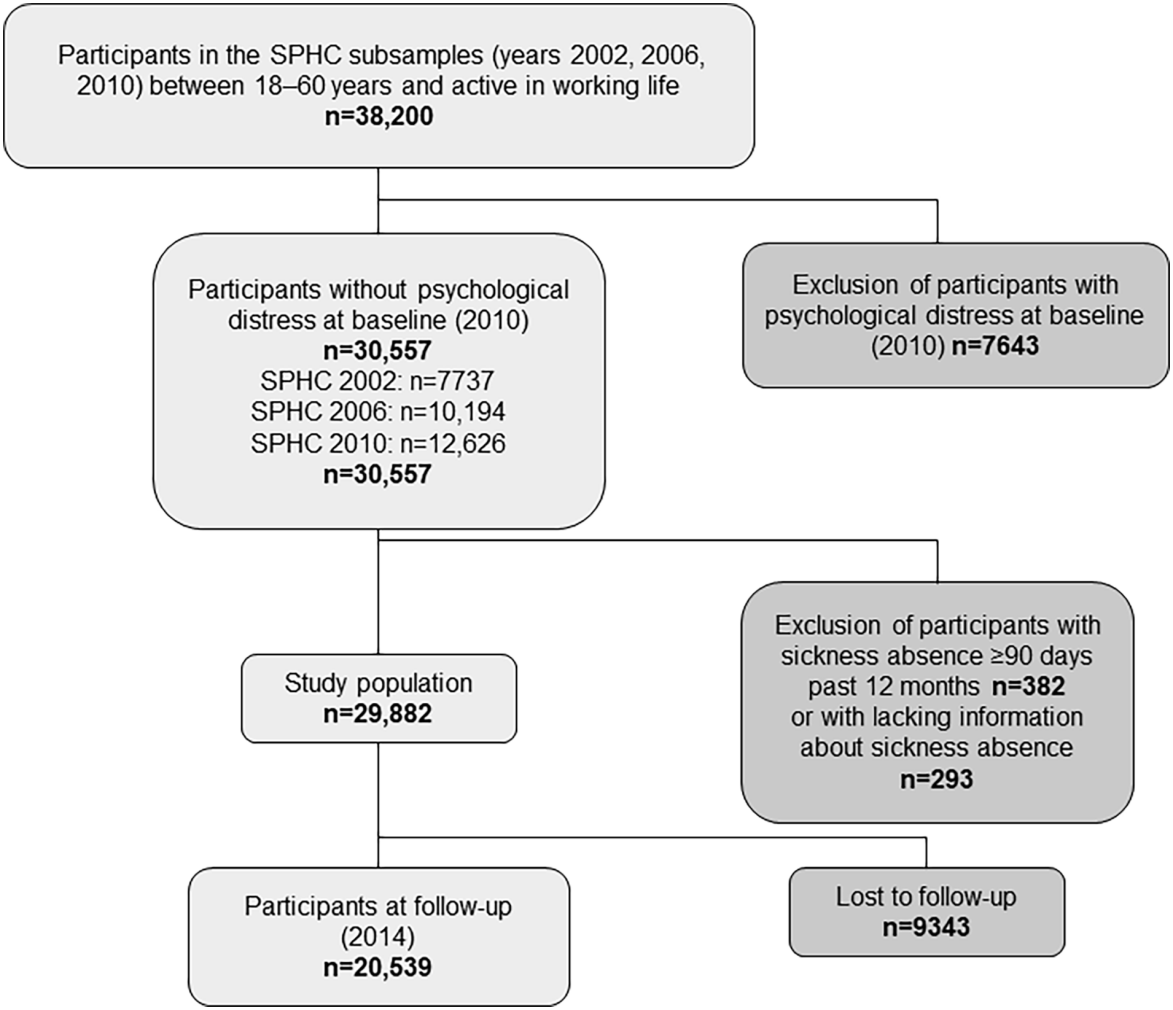
Flowchart of participants from baseline to follow-up. SPHC, Stockholm Public Health Cohort.

The total study population at baseline was 29,882, as presented in Figure 1. The follow-up rate was 69%, hence 20,539 participants were successfully followed in 2014. Table II displays a detailed description of baseline characteristics stratified by good and poor work ability. The mean age was 45.6 years and 55% were women. Most participants were intermediate non-manual workers and employed/self-employed professionals and 22% had previous long-term illness. Out of the included participants, 92% reported good work ability at baseline.

**Table II.** Characteristics of study sample stratified by work ability (*n*=29,882).

Variables^c^	All		Good work ability^a^		Poor work ability^b^	
	*n*	%	*n*	%	*n*	%
All	29,882	100	27,373	92	2509	8
Age, mean years (SD)	45.6 (10)		45.3 (10)		46.6 (10)	
Women	16,426	55	14,898	54	1528	61
Married	15,487	52	14,255	52	1232	49
Living alone	4136	14	3711	14	425	17
Missing	109	<0.5	95	<0.5	14	<0.5
Socioeconomic status						
Unskilled/semiskilled workers	3494	12	2909	11	585	25
Skilled workers	2977	11	2573	10	404	17
Assistant non-manual workers	3856	14	3587	14	269	11
Intermediate non-manual workers	8218	29	7657	29	561	24
Employed/self-employed professionals	7111	25	6786	26	325	14
Self-employed other than professionals	2794	10	2566	10	228	10
Missing	1432	5	1295	5	137	5
Body mass index, mean (SD)	25.6 (7)		25.0 (5)		25.9 (8)	
Missing	547	2	480	2	67	2
Daily smoking	2959	10	2537	9	422	17
Missing	183	1	158	1	25	1
Leisure-time physical activity						
No/low	21,709	73	19,719	72	1990	80
Moderate	7114	24	6665	24	449	18
High	976	3	924	3	52	2
Missing	83	<0.5	65	<0.5	18	1
Sleep disturbances	7228	25	6069	23	1159	48
Missing	518	2	435	2	83	3
Neck/back pain past 6 months	18,759	63	16,760	62	1999	80
Missing	190	1	173	1	17	1
Long-term illness	6396	22	5266	19	1130	46
Missing	267	1	227	1	40	2
Baseline (2010), subsamples						
SPHC 2002	7589	25	7013	26	576	23
SPHC 2006	10,011	34	9230	34	781	31
SPHC 2010	12,282	41	11,130	41	1152	46
Follow-up (2014)	20,539	69	18,964	69	1575	63

SPHC: Stockholm Public Health Cohort.

a Good work ability defined as answering ‘very good’ or ‘rather good’ on the Work Ability Index about self-perceived physical and mental work ability.

b Poor work ability defined as answering ‘moderate’, ‘rather poor’ or ‘poor’ on the Work Ability Index about self-perceived physical and mental work ability.

c Missing states variables with internal missing values, other variables include all participants.

Crude and adjusted associations between work ability and PD are presented in Table III. At follow-up, 2543 participants (12%) had developed PD according to the GHQ-12. Of those with poor work ability, 14% (*n*=360) developed PD during the study period. The adjusted association between poor work ability and PD yielded a RR of 1.8 (95% CI 1.6–2.0). Thus, reporting poor work ability at baseline was associated with an almost doubled RR of PD at follow-up after 4 years, compared to reporting good work ability at baseline. The attrition analysis showed that of those participants lost to follow-up, 10% (*n*=934) reported poor work ability according to the WAI. The corresponding prevalence for those successfully followed was 8% (*n*=1575).

**Table III.** Associations between work ability and the incidence of psychological distress.^a^

	Cases/total	Crude		Adjusted^b^	
		RR	95% CI	RR	95% CI
Good work ability^c^	2183/18,964	1		1	
Poor work ability^d^	360/1575	2.0	1.8–2.2	1.8	1.6–2.0

CI: confidence interval; RR: rate ratio.

a Psychological distress defined as scoring 4 or more on the General Health Questionnaire 12.

b Adjusted for age, sleep disturbances, neck/back pain past 6 months and cohort subsample.

c Good work ability defined as answering ‘very good’ or ‘rather good’ on the Work Ability Index about self-perceived physical and mental work ability.

d Poor work ability defined as answering ‘moderate’, ‘rather poor’ or ‘poor’ on the Work Ability Index about self-perceived physical and mental work ability.

## Discussion

This prospective study assessed the role of self-perceived physical and mental work ability for the incidence of PD in a Swedish working population. The results indicate that self-perceived poor physical and/or mental work ability in relation to job demands is associated with an almost doubled RR of developing PD over time, compared to a good work ability.

To the best of our knowledge, this is the first study to investigate poor work ability as a potential risk factor for PD. Previous studies in occupational health research have identified other psychosocial work-related risk factors, including high job demands, low support, bullying [5, 6] and job strain [6, 7] for depression and anxiety symptomatology [5, 6] as well as for clinically diagnosed conditions [7]. Leijon et al. (2017) found PD to be a risk factor for future poor work ability in a longitudinal study of Swedish workers [18]. In this study, however, PD was instead the outcome of interest in relation to work ability and not the exposure.

The results of this study are of societal as well as individual importance. A Swedish report states that sick leave resulting from mental health problems on average is longer compared to sick leave from other diagnoses [2]. Furthermore, although individuals with poor work ability may be able to perform work which is less physically demanding, it is reasonable to assume that poor work ability related to mental demands hampers the ability to perform work which is also less demanding. Also, the possibility to start working after sick leave due to mental health problems is poorer than for other diagnoses, adding to the present study’s result and stressing the importance of acknowledging mental health in occupational settings, maybe even to a greater extent than previously. High comorbidity between PD and conditions such as spinal pain [26] and cardiovascular disease, among others, is another incentive for increasing the awareness of mental health, which is also of high priority in the United Nations’ Millennium Development Goals striving towards sustainable health for everyone [27]. The results of this study emphasise the importance of future studies assessing the role of work ability among workers in occupational settings for preventing future PD.

This population-based study has a substantial sample size, covering residents in the largest county in Sweden. Extensive questionnaires enabled a careful consideration of potential confounders even though there is a risk of unmeasured and residual confounding (e.g. job loss, job dissatisfaction, etc.) that may entail an under- or overestimation of associations. Moreover, selection bias may have an impact on the results if loss to follow-up was associated both with the prevalence of poor work ability (exposure) and the incidence of PD (outcome). In this case, however, the prevalence of the exposure was similar among successfully followed participants and drop-outs (10% and 8%, respectively), indicating that selection bias is not a big threat.

The exposure and outcome were assessed with stable instruments of high validity previously used in occupational research. Work ability was assessed with only one subscale of the WAI, and the exposure may hence be prone to non-differential misclassification. Nonetheless, this subscale from the WAI has been validated both against the full WAI (i.e. all seven subscales) as well as for sickness absence [28]. If misclassification biases the results, this would have led to diluted associations. Furthermore, changing jobs during the study period may also result in changed perceived work ability. Measuring job changes during the study period would possibly result in a more accurate measure of the longitudinal association with PD; however, this was not assessed in this study and cannot be taken into consideration.

To avoid morbidity influencing participants’ judgement of work ability for illness not related to PD, those with sickness absence for more than 90 days during the past 12 months were excluded from the cohort. The GHQ-12 is a non-specific screening measure of PD often used in primary care, and is not an instrument used for diagnosing mental disease [29]. In this study, only participants scoring 2 or less on the GHQ-12 at baseline were included to be able to study the risk of PD rather than the prognosis.

The outcome PD was defined as scoring 4 or more on the GHQ-12, which previously has been suggested to be the best cut-off for identifying PD with this instrument [23]. Scoring 4 or more on the GHQ-12 corresponds to moderate/severe PD, which may reflect a condition that is more than subclinical, hence motivating a more thorough mental health examination.

The aim of the study was to investigate the incidence of PD in a healthy cohort. Rai et al. (2012) concluded that also milder forms of PD (scoring 1–2 on the GHQ-12), corresponding to ‘good mental wellbeing’ in Swedish surveys that form the basis of public mental health policies, were associated with receiving a disability pension after 5 years [30]. The findings by Rai et al. (2012) may indicate that this study has examined the prognosis rather than the incidence of PD and is a rationale for acknowledging also mild PD in future research in occupational settings [30]. Moreover, it is reasonable to assume that the two different dimensions of work ability measured in this study (i.e. physical and mental, respectively) differ in their contribution to PD, which motivate future research studying these separately rather than collectively. By doing this, it is possible to identify the demands of highest importance for developing PD and implement future preventive strategies in occupational settings accordingly.

In conclusion, this study adds new knowledge to the field of occupational research by indicating that poor work ability is associated with a higher incidence of future PD compared to good work ability.

## References

[1] James S. L. Geleijnse J. M. (2018). Global, regional, and national incidence, prevalence, and years lived with disability for 354 diseases and injuries for 195 countries and territories, 1990 -2017: a systematic analysis for the Global Burden of Disease Study 2017. The Lancet 392(10159): 1789–1858. 10.1016/S0140-6736(18)32279-7 PMC6227754 30496104

[2] Försäkringskassan (2018). Svar på regeringsuppdrag – Uppföljning på sjukfrånvarons utveckling 2018. Socialförsäkringsrapport 002671–2018. https://www.forsakringskassan.se/wps/wcm/connect/d3d2d056-0ae7-46d9-b350-ac87e4696f1c/rapport-uppfoljning-av-sjukfranvarons-utveckling-2018-svar-pa-regeringsuppdrag-dnr-002671-2018.pdf?MOD=AJPERES&CVID=

[3] Cuijpers P Smit F. Subthreshold depression as a risk indicator for major depressive disorder: a systematic review of prospective studies. Acta Psychiatr Scand 2004;109:325–331. 15049768 10.1111/j.1600-0447.2004.00301.x

[4] Shankman SA Lewinsohn PM Klein DN , et al. Subthreshold conditions as precursors for full syndrome disorders: a 15-year longitudinal study of multiple diagnostic classes. J Child Psychol Psychiatry 2009;50:1485–1494. 19573034 10.1111/j.1469-7610.2009.02117.x PMC2804772

[5] Harvey SB Modini M Joyce S , et al. Can work make you mentally ill? A systematic meta-review of work-related risk factors for common mental health problems. Occup Environ Med 2017;74:301–310. 28108676 10.1136/oemed-2016-104015

[6] Theorell T Hammarström A Aronsson G , et al. A systematic review including meta-analysis of work environment and depressive symptoms. BMC Public Health 2015;15:738. 26232123 10.1186/s12889-015-1954-4 PMC4522058

[7] Madsen IEH Nyberg ST Magnusson Hanson LL , et al. Job strain as a risk factor for clinical depression: systematic review and meta-analysis with additional individual participant data. Psychol Med 2017;47:1342–1356. 28122650 10.1017/S003329171600355X PMC5471831

[8]] TenglandPA. The concept of work ability. J Occup Rehabil2011;21:275–285.2105280710.1007/s10926-010-9269-x

[9] Alavinia SM de Boer AG van Duivenbooden JC , et al. Determinants of work ability and its predictive value for disability. Occup Med 2009;59:32–37. 10.1093/occmed/kqn148 19073989

[10] Ilmarinen J Tuomi K Klockars M. Changes in the work ability of active employees over an 11-year period. Scand J Work Environ Health 1997;23(Suppl.1):49–57. 9247995

[11] Tuomi K Ilmarinen J Klockars M , et al. Finnish research project on aging workers in 1981–1992. Scand J Work Environ Health 1997;23(Suppl.1):7–11. 9247990

[12] Notenbomer A Groothoff JW van Rhenen W , et al. Associations of work ability with frequent and long-term sickness absence. Occup Med 2015;65:373–379. 10.1093/occmed/kqv052 25964509

[13] Kinnunen U Natti J. Work ability score and future work ability as predictors of register-based disability pension and long-term sickness absence: a three-year follow-up study. Scand J Public Health 2018;46:321–330. 29212430 10.1177/1403494817745190

[14] Ohta M Higuchi Y Kumashiro M , et al. Decrease in Work Ability Index and sickness absence during the following year: a two-year follow-up study. Int Arch Occup Environ Health 2017;90:883–894. 28795227 10.1007/s00420-017-1251-x

[15] Lundin A Kjellberg K Leijon O , et al. The association between self-assessed future work ability and long-term sickness absence, disability pension and unemployment in a general working population: a 7-year follow-up study. J Occup Rehabil 2016;26:195–203. 26319413 10.1007/s10926-015-9603-4

[16] Ebener M Hasselhorn HM . Validation of Short Measures of Work Ability for Research and Employee Surveys. Int J Environ Res Public Health 2019 Sep 12;16(18):3386. 10.3390/ijerph16183386 PMC6765804 31547466

[17] van den Berg TI Elders LA de Zwart BC , et al. The effects of work-related and individual factors on the Work Ability Index: a systematic review. Occup Environ Med 2009;66:211–220. 19017690 10.1136/oem.2008.039883

[18] Leijon O Balliu N Lundin A , et al. Effects of psychosocial work factors and psychological distress on self-assessed work ability: a 7-year follow-up in a general working population. Am J Ind Med 2017;60:121–130. 27779327 10.1002/ajim.22670

[19] Svensson AC Fredlund P Laflamme L , et al. Cohort profile: the Stockholm Public Health Cohort. Int J Epidemiol 2013;42:1263–1272. 23042793 10.1093/ije/dys126

[20] Lundin A Hallgren M Theobald H , et al. Validity of the 12-item version of the General Health Questionnaire in detecting depression in the general population. Public Health 2016;136:66–74. 27040911 10.1016/j.puhe.2016.03.005

[21] de Zwart BC Frings-Dresen MH van Duivenbooden JC. Test–retest reliability of the Work Ability Index questionnaire. Occup Med 2002;52:177–181. 10.1093/occmed/52.4.177 12091582

[22] Radkiewicz P Widerszal-Bazyl M. Psychometric properties of Work Ability Index in the light of comparative survey study. Int Congr 2005;1280:304–309.

[23] Lundin A Ahs J Asbring N , et al. Discriminant validity of the 12-item version of the general health questionnaire in a Swedish case-control study. Nord J Psychiatry 2017;71:171–179. 27796153 10.1080/08039488.2016.1246608

[24] Pevalin DJ. Multiple applications of the GHQ-12 in a general population sample: an investigation of long-term retest effects. Soc Psychiatry Psychiatr Epidemiol 2000;35:508–512. 11197926 10.1007/s001270050272

[25] Montazeri A Harirchi AM Shariati M , et al. The 12-item General Health Questionnaire (GHQ-12): translation and validation study of the Iranian version. Health Qual Life Outcomes 2003;1:66. 14614778 10.1186/1477-7525-1-66 PMC280704

[26] Paanalahti K Holm LW Magnusson C , et al. The sex-specific interrelationship between spinal pain and psychological distress across time in the general population. Results from the Stockholm Public Health Study. Spine J 2014;14:1928–1935. 24262854 10.1016/j.spinee.2013.11.017

[27] Prince M Patel V Saxena S , et al. No health without mental health. Lancet 2007;370:859–877. 17804063 10.1016/S0140-6736(07)61238-0

[28] Lundin A Leijon O Vaez M , et al. Predictive validity of the Work Ability Index and its individual items in the general population. Scand J Public Health 2017;45:350–356. 28385066 10.1177/1403494817702759

[29] Lundin A Dalman C. Psykisk ohälsa mätt med the General Health Questionnaire. En validering i Stockholms län: Centrum för epidemiologi och samhällsmedicin, Stockholms läns landsting; 2020. Rapport 2020:5.

[30] Rai D Kosidou K Lundberg M , et al. Psychological distress and risk of long-term disability: population-based longitudinal study. J Epidemiol Community Health 2012;66:586–592. 21422028 10.1136/jech.2010.119644

